# Supplementary material for: The scaling structure of the global road network
Source: R Soc Open Sci. 2017 Oct 18;4(10):170590. doi: 10.1098/rsos.170590 (PMC5666254; doi:10.1098/rsos.170590)
Supplement: Methodological details, results and data visualization [file rsos170590supp1.pdf]

The scaling structure of the global road network. SUPPLEMENTARY MATERIAL

Emanuele Strano,[1,2,3] Andrea Giometto,[4,5] Saray Shai,[6]
Enrico Bertuzzo,[7,5] Peter J. Mucha,[6] and Andrea Rinaldo[5,8]

[1]*Department of Civil and Environmental Engineering, Massachusetts Institute of Technology (MIT), Cambridge, MA 02139, USA*

[2]*German Aerospace Center (DLR), German Remote Sensing Data Center (DFD), Oberpfaffenhofen, D-82234 Wessling, Germany*

[3]*Laboratory of Geographic Information Systems (LaSig), Polytechnical School Of Lausanne (EPFL), Lausanne, CH-1015, Switzerland*

[4]*Department of Physics, Harvard University, MA-02138 Cambridge, United States*

[5]*Laboratory of Ecohydrology, École Polytechnique Fédérale Lausanne (EPFL), Lausanne, CH-1015, Switzerland*

[6]*Department of Mathematics, University of North Carolina, Chapel Hill, NC 27599, USA*

[7]*Department of Environmental Sciences, Informatics and Statistics, University Ca' Foscari Venice, Venezia Mestre, 30170, Italy*

[8]*Department of Civil, Environmental and Architectural Engineering, University of Padova, Padova, 35131, Italy*

(Dated: August 10, 2017)

DATA PREPARATION AND DATA FUSION

The general idea here is to transfer the land-use information, represented by continuous values, onto the road network, which is represented by lines vectorial geometry. The three land uses are urban, crop-land and semi-natural. The overall output is a road network in which each road is labeled of the land-use class it belongs to. In order to do so, a series of spatial operations have been performed, mainly in the ArchMap, Qgis and Python environments. The data preparation schema is presented in Fig. 1. The schema is organized in the data preparation phases, followed by a spatial join, which assign each road to a land use.

Urban footprint

Phase one (Fig. 1A and D) extracted the global urban footprint from the nighttime light layer (DMSP-OLS). Nighttime light is a continuous layer at 30arcsec (1km at the equator) pixel unit with

values ranging between 0 and 63. Each value represents the annual average brightness. The data were produced by NOAA's National Geophysical Data Center and are publicly available. DMSP-OLS data were collected by the US Air Force Weather Agency. DMSP-OLS have been widely used to characterize the urban footprint and urban evolution at global scales [1, 2]. To extract an urban settlements, a Jenks clustering algorithm has been applied [3]. A detailed visualization of the resulting classification is provided in Fig. 4A, Fig. 2 and Fig. 3. An alternative classification has been performed dividing the illumination range into five equally distant bins (Fig. 4B). Given the sharp differences in illumination level between urban and peri-urban areas, the results of the two methodologies almost overlap to the same urban mask and thus to same road length distributions.

Cropland

The global IIASA-IFPRI [4] cropland percentage map for the baseline year 2005 has been developed by integrating a number of individual cropland maps at global to regional to national scales, including existing global land cover maps such as GlobCover 2005 and MODIS v.5, regional maps such as AFRICOVER, and national maps from mapping agencies and other organizations. IIASA-IFPRI is a public data. A visualization of cropland mask is provided in Fig. 1B, Fig. 2A and Fig. 3

Road network and land-use labeling method

The GRN data set was acquired from DeLorme (Yarmouth, ME, USA). It contains an updated version of all major roads on earth in 2014. The network is organized into four hierarchies: primary roads with limited access (H1), primary roads with non-limited access (H2), minor and secondary roads (H3), and local non-urban roads (H4). As shown in Fig. 2, the road network data set does not contain minor urban roads. The topology of the road network has been corrected using Archmap software and ad hoc Python scripts for the purpose of joining connected roads at junctions with only two roads and to remove small road links representing highway ramps and cross roads intersections, which are not representative of any fragmentation process and are potential noise for the statistical analysis. Roads shorter than 100 m cover only $\approx 0.03\%$ of the total road length; these roads, as confirmed by an extensive and scrupulous inspection of the GLR dataset, appear to be highway ramps or road segments for large road junctions and were therefore excluded from our analyses. Coupling the GNR with two global land-use inventories, road have been classified

into three categories: urban roads, i.e. roads that entirely belong to urban areas, crop-land roads: i.e. road that intersect or belong to crop-land areas, and semi-natural roads, i.e. road that are completely free of direct urban or cropland use. Fig. 2,3 and 4 illustrates the final classification.

URBAN ROAD NETWORK EVOLUTION

We observe that the distribution of urban patch's areas has a power-law tail $A^{-\alpha}$ with exponent $\alpha \simeq 2$. Specifically, the mean exponent estimated via maximum likelihood in 16 night-light (1997-2012) censuses is $\alpha = 2.05 \pm 0.01$ and the p-values computed according to the method outlined in Clauset et al., SIAM Review 51(4), 2009 are larger than 0.1 for each census, thus urban areas are distributed according to a power-law (note that the hypothesis is rejected if $p < 0.1$ in this hypothesis test, unlike in usual statistics). Furthermore, we observe that the total number of urban areas and the maximum urban area increase linearly with the census. The increase of the maximum urban area with time is a by-product of the increase of the number of urban patches and is compatible with a power-law distribution of urban areas. In fact, because the distribution of urban areas is the power-law $p(A) = A_{\min} A^{-2}$, the expected maximum urban area in N samples (i.e., N is the total number of urban areas in a census) is $A_{\max} = A_{\min} N^{1/(\alpha-1)} = A_{\min} N$ and the mean urban area is thus given by:

$$\langle A \rangle = A_{\min} \log \left(\frac{A_{\max}}{A_{\min}} \right) = A_{\min} \log N. \tag{1}$$

Panels e and f in Fig. 5 show the scaling of $\langle A \rangle$ and $A_{\max}$ in the various censuses, which are characterized by different values of N. The mean area $\langle A \rangle$ increases very weakly with N, and accordingly the slope of the best fit of $\log\langle A \rangle$ vs $\log N$ (red line in panel e) is compatible with zero (0.23 ± 0.12, mean$\pm$SE). The slope of the best fit of $\log A_{\max}$ vs $\log N$ (red line in panel f) is compatible with unity (1.31 ± 0.27, mean$\pm$SE). These scalings are compatible with a power-law distribution of urban areas with exponent $\alpha = 2$ and thus we interpret the various censuses as draws from such a power-law distribution. We now ask the following question: how many kilometers of paved roads will be necessary to support a 10% increase in the total urban area?

Assuming that the distribution of urban areas will remain unchanged, as observed in the censuses plotted in Fig. 5, an increase in the total urban area is due to an increase in the number of urban patches. The average total length in a sample of N urban patches is $L_{tot} = N \int_{A_{\min}}^{A_{\max}} \langle L|A \rangle p(A) dA$, where $\langle L|A \rangle$ is the average total road length in an urban patch of area A, which increases linearly with the urban area A as $\langle L|A \rangle = cA$ with constant c, see Fig. 2C of the main text. The best-fit

estimate of c obtained by fitting $\ln\langle L|A\rangle$ to $\ln A$ (red points in Fig. 2C) is $c^{-1} = (1.8 \pm 0.1) \cdot 10^3$

$$L_{tot} = N \int_{A_{\min}}^{A_{\max}} \langle L|A\rangle p(A) dA = cNA_{\min} \ln \frac{A_{\max}}{A_{\min}}. \tag{2}$$

To answer the question above, we need to express the number of urban patches as a function of the total urban area, where the latter is given by $A_{tot} = N\langle A\rangle = A_{\min} N \log N$ (see Eq. 1). Thus, N as a function of A is given by $N = A_{tot}/A_{\min} W^{-1}(A_{tot}/A_{\min})$, where W is the Lambert W function. We can now substitute this expression for N in Eq. 2 and use the scaling of $A_{\max}$ with N to obtain L_{tot} as a function of A_{tot}:

$$L_{tot} = cA_{tot}, \tag{3}$$

therefore L_{tot} increases linearly with the total urban area A_{tot}.

I. AN ATLAS OF GLOBAL ROAD NETWORK

Figures 6–12 depict different geographical parts of the GRN.

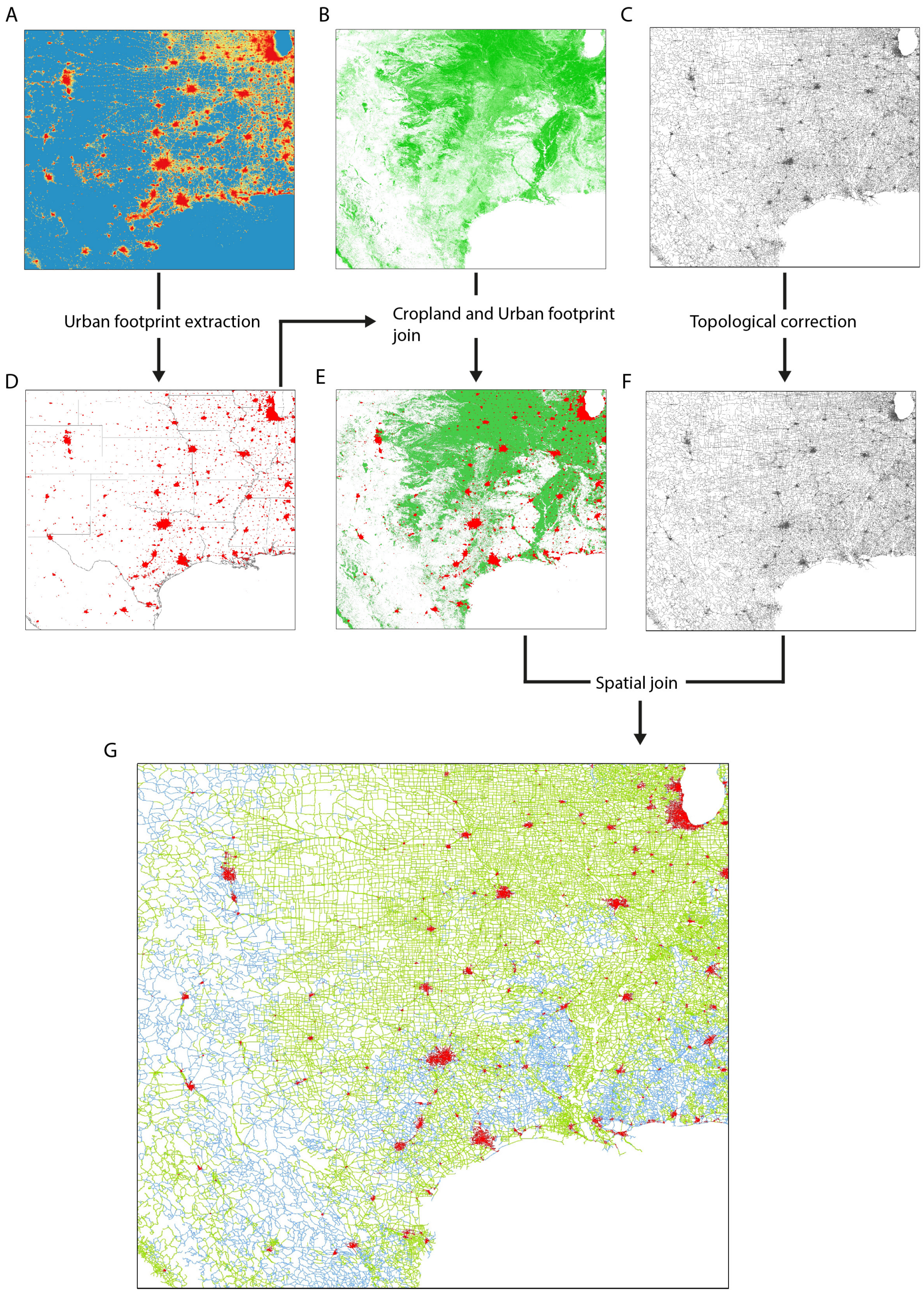

FIG. 1. Global roads network data preparation. (A) Nighttime light data (B) Global cropland layer. (C) Global road network. (D) Urban footprint mask extracted with Jenks clustering algorithm. (E) Cropland and urban footprint mask. (F) Corrected global road network, in this case the corrections are not visible. (G) Final road network divided in three classes.

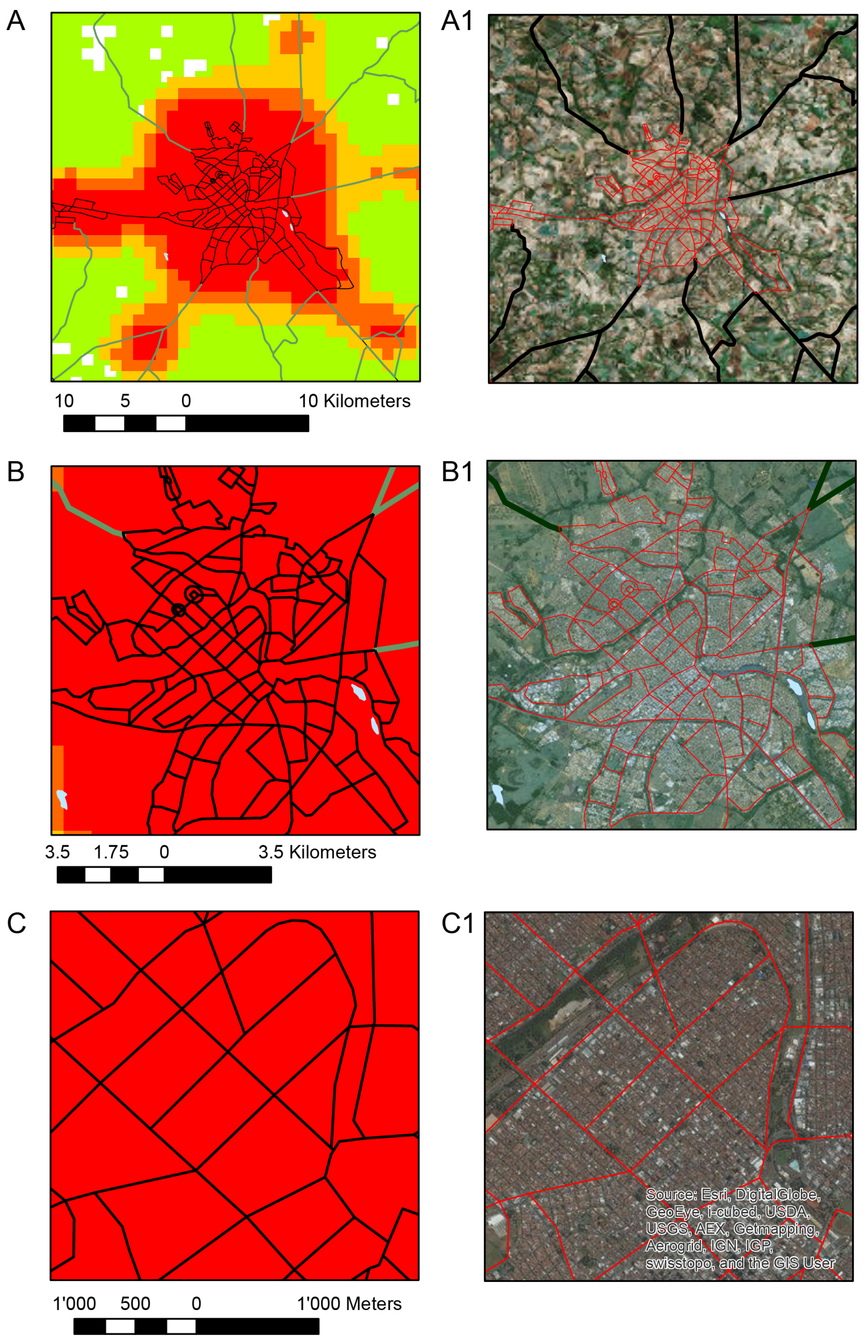

FIG. 2. Details on urban land use classification and road network labeling. Images in the left column show the data used for the classification, in balck urban roads and in green crop-land road. All images in the right column show satellite images on the same areas, in red urban roads and in black the crop-land roads. In all figures red is urban and green is cropland. Roads have been labeled as urban if the entirely belong to an illuminated area, while if they intersect a cropland area they have been labeled as crop-land. Comparing A with A1 and B to B1 is possible to observe that road labels properly overlap the land-use. C and C1 show the scale of road network in cities, in particular one can note that urban road cannot be used to describe block size.

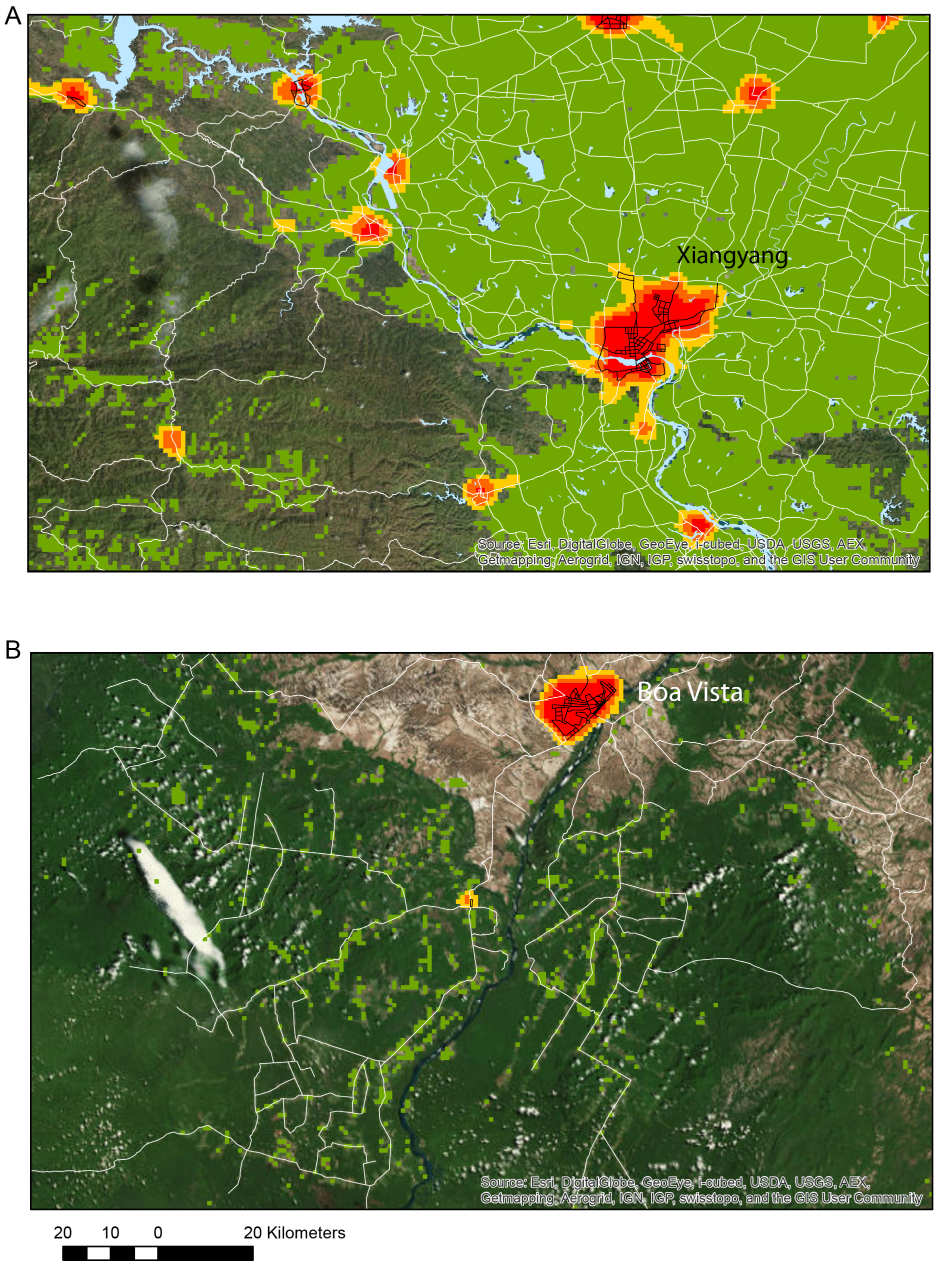

FIG. 3. A detailed visualization of crop-land roads and crop-land patches in two areas. In A and B crop-land are white. It is possible to observe that small crop-land units are located in natural areas while compact cropland regions are located around cities. It is also evident that a single roads segment can serve more that a cropland unit.

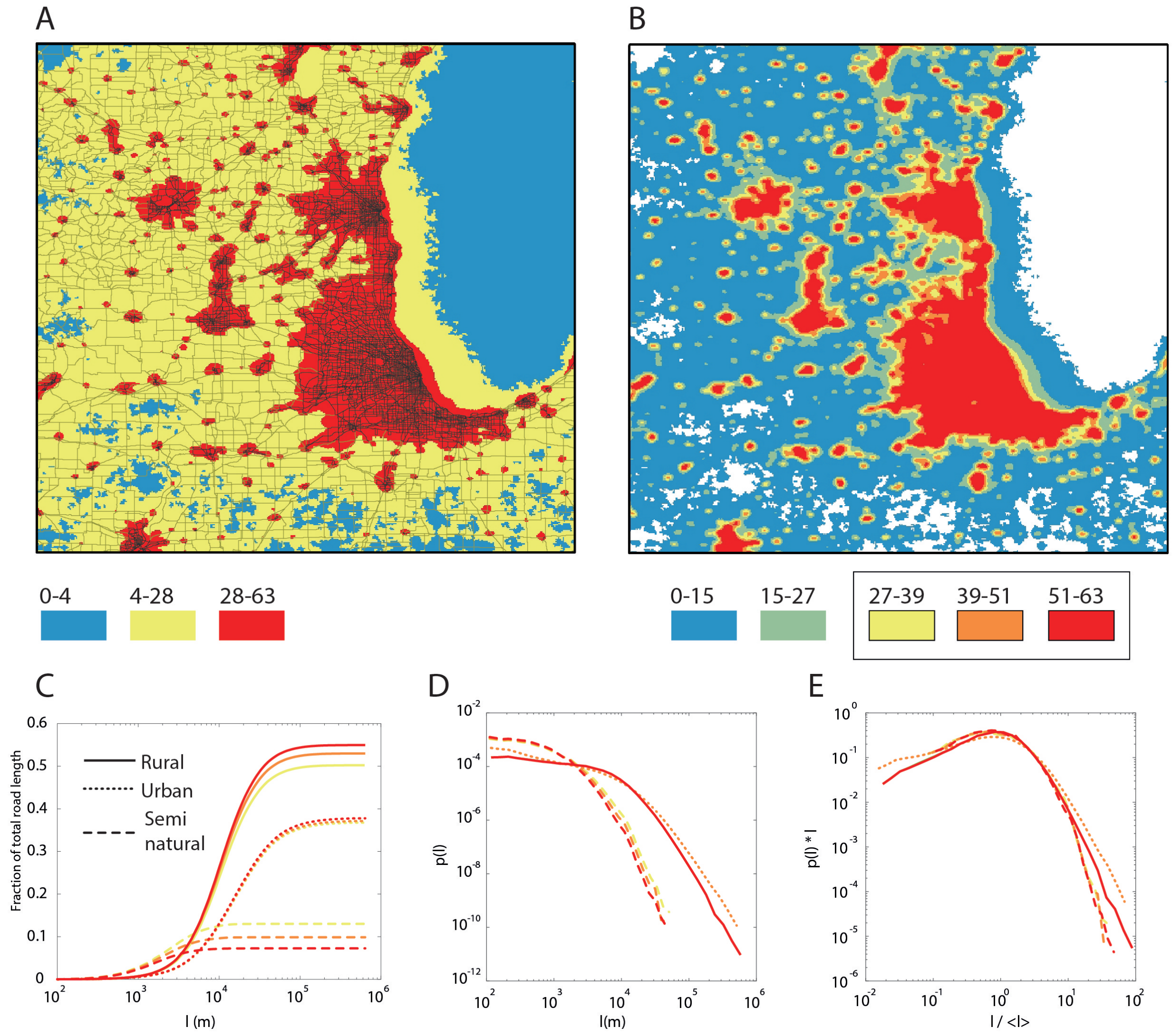

FIG. 4. (A) The classification of urban areas with Jenks algorithm used for the main results of analysis: each color represents a range of illumination as reported in the legend. For our analysis we used only the dense part represented in red. A linear bin classification has been also tested (B). Panels A and B have been extracted from the same area around Chicago metropolitan area (Illinois, US). In B, roads have been not represented. We can observe that A and B represent the urbanized areas. The same analysis as for Fig. 1 in the main text has been reproduced in C, D and E. Colors in C, D and E represent respectively the three denser classes as illustrated in B. We can observe that main scaling behavior is not affected by different density or classification. It is important to note that roads in A have been extracted if fully belonging to the red areas, while for B roads have been trimmed to the edge of urban areas, thus producing smaller segments. Smaller segment abundances in B might have produced the deviation for scaling in the tail of the distribution in E.

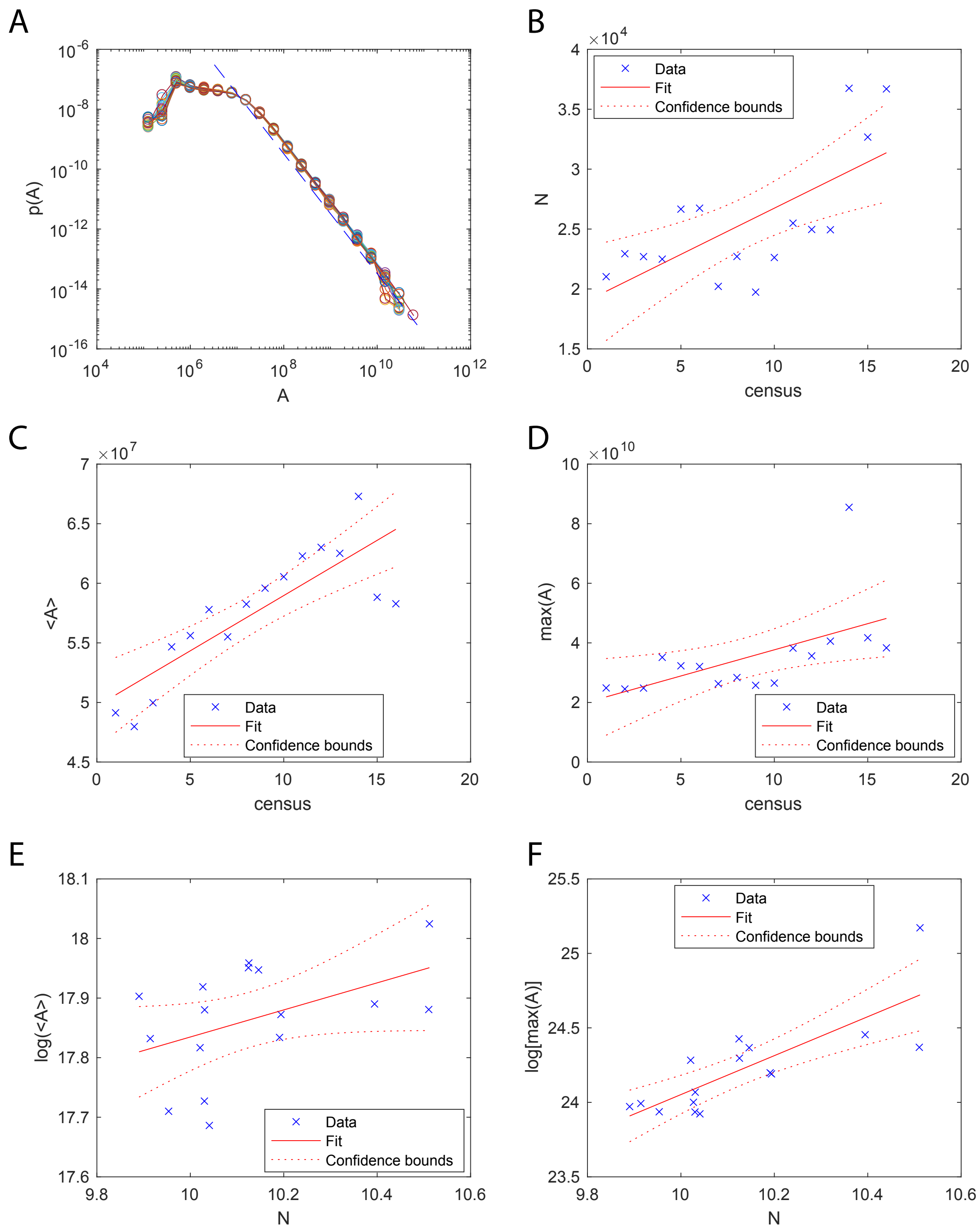

FIG. 5. A) Distribution of urban areas in 16 night-light censuses (colored solid curves). The dashed line is a power-law $A^{-\alpha}$ with exponent $\alpha = 2$. B) Number of urban areas in each census. C) Mean urban area in each census. D) Maximum urban area in each census. E) Mean urban area vs number of urban areas. F) Maximum urban area vs number of urban areas.

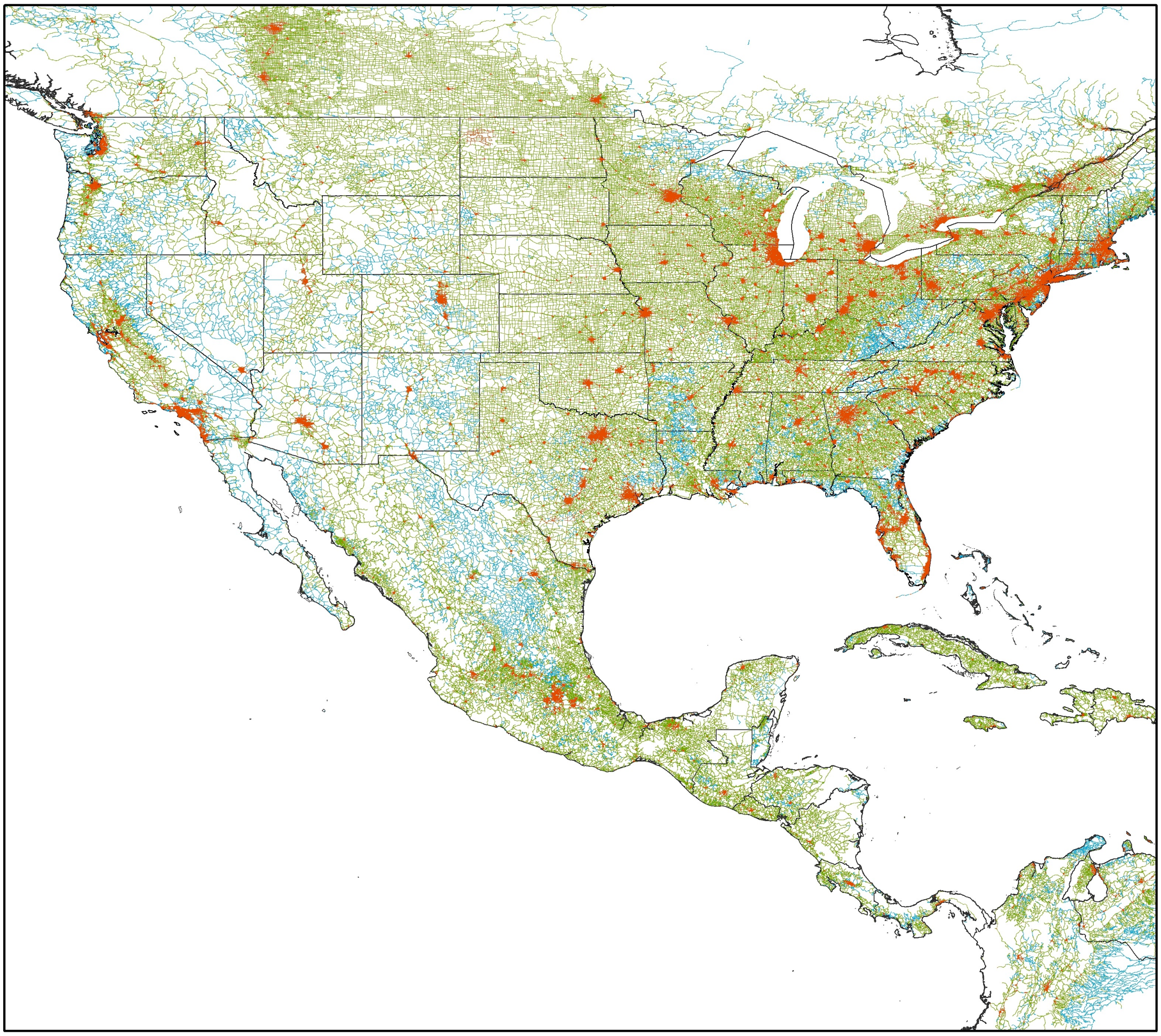

FIG. 6. Visualization of North and Central America GRN.

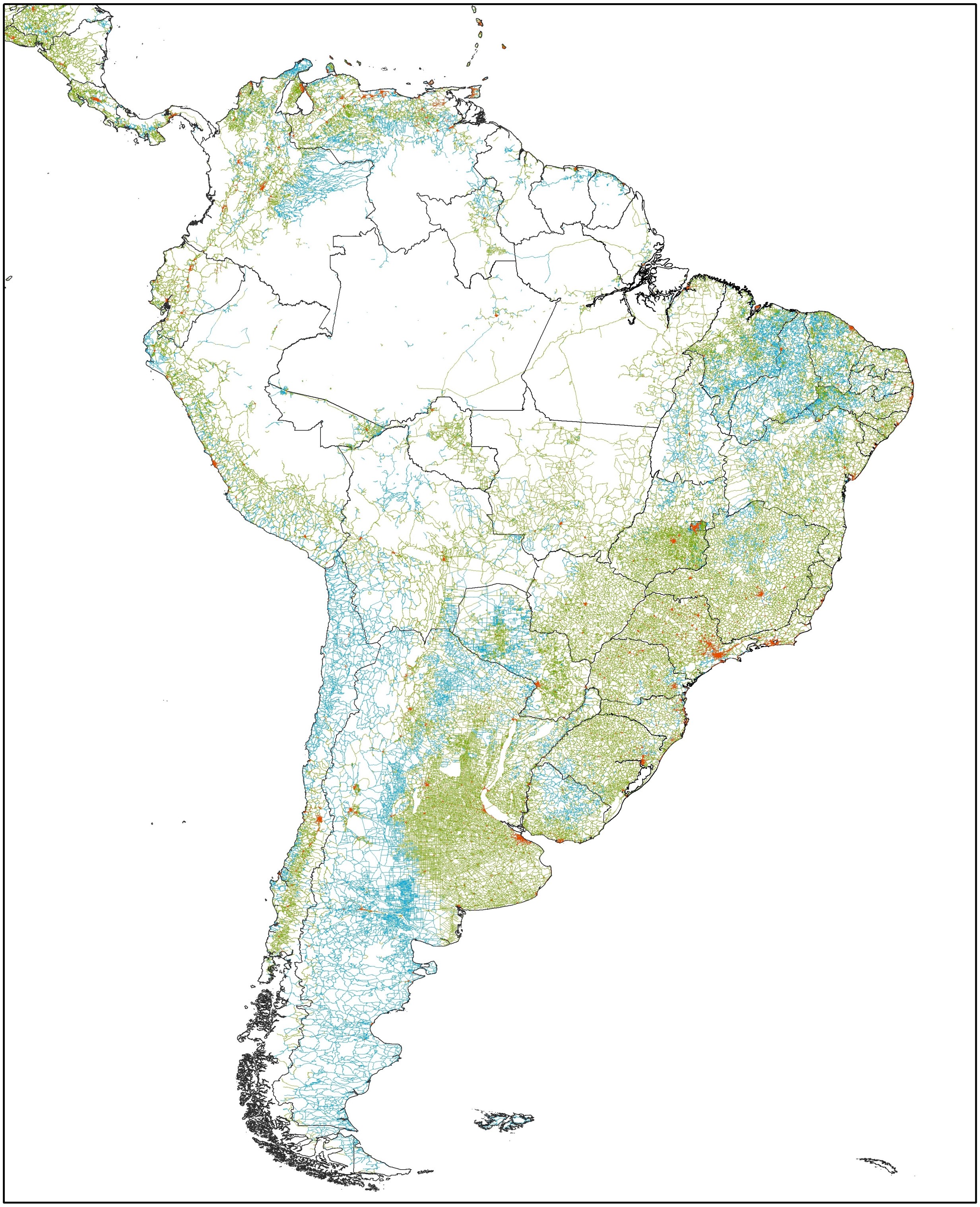

FIG. 7. South America GRN. We observe that longer roads belonging to the Amazon are classified here as agricultural roads. Such classification might appear counter-intuitive, but it illustrates a condition in which forest erosion is driven by agricultural expansion.

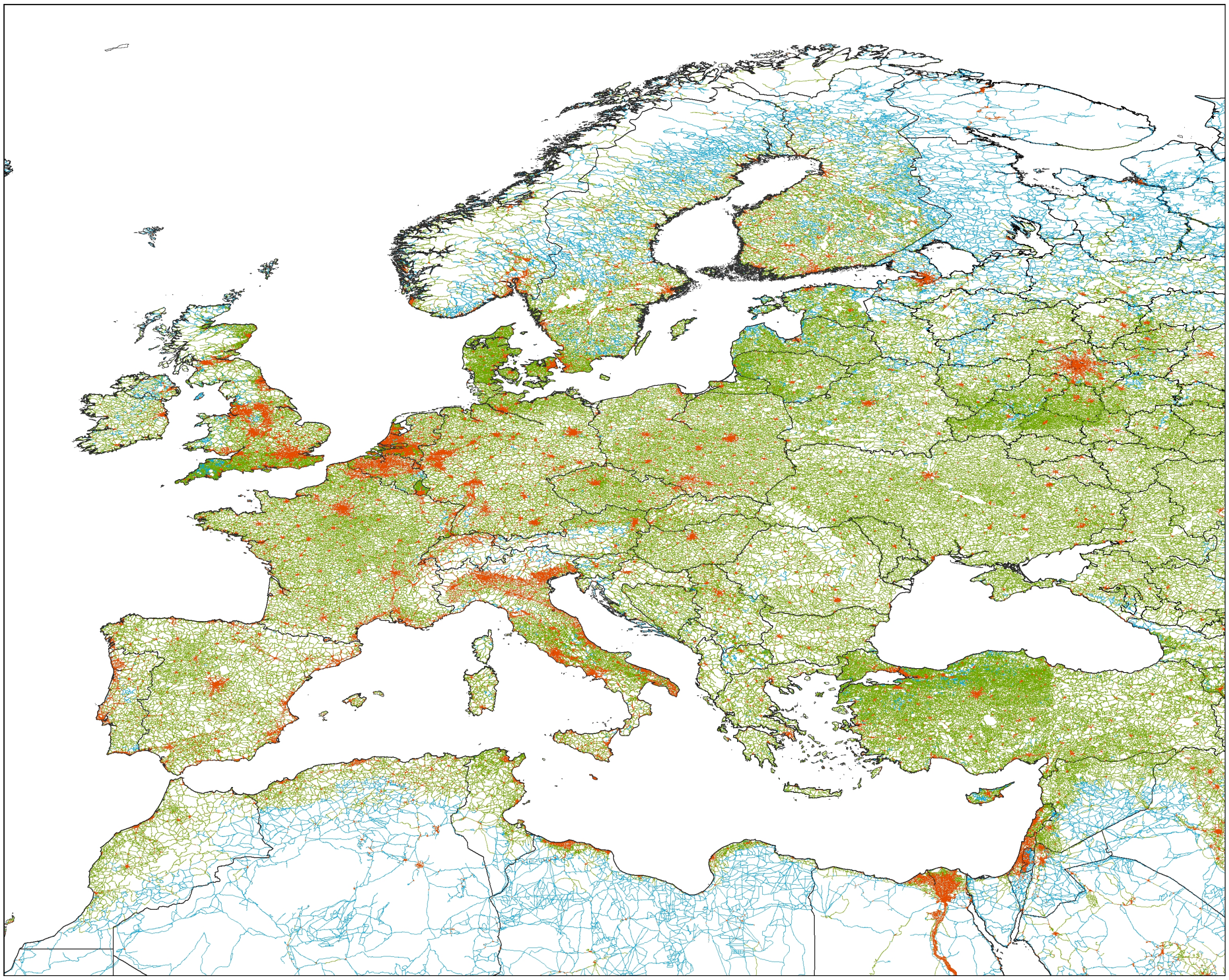

FIG. 8. The GRN in Europe and the Mediterranean Basin.

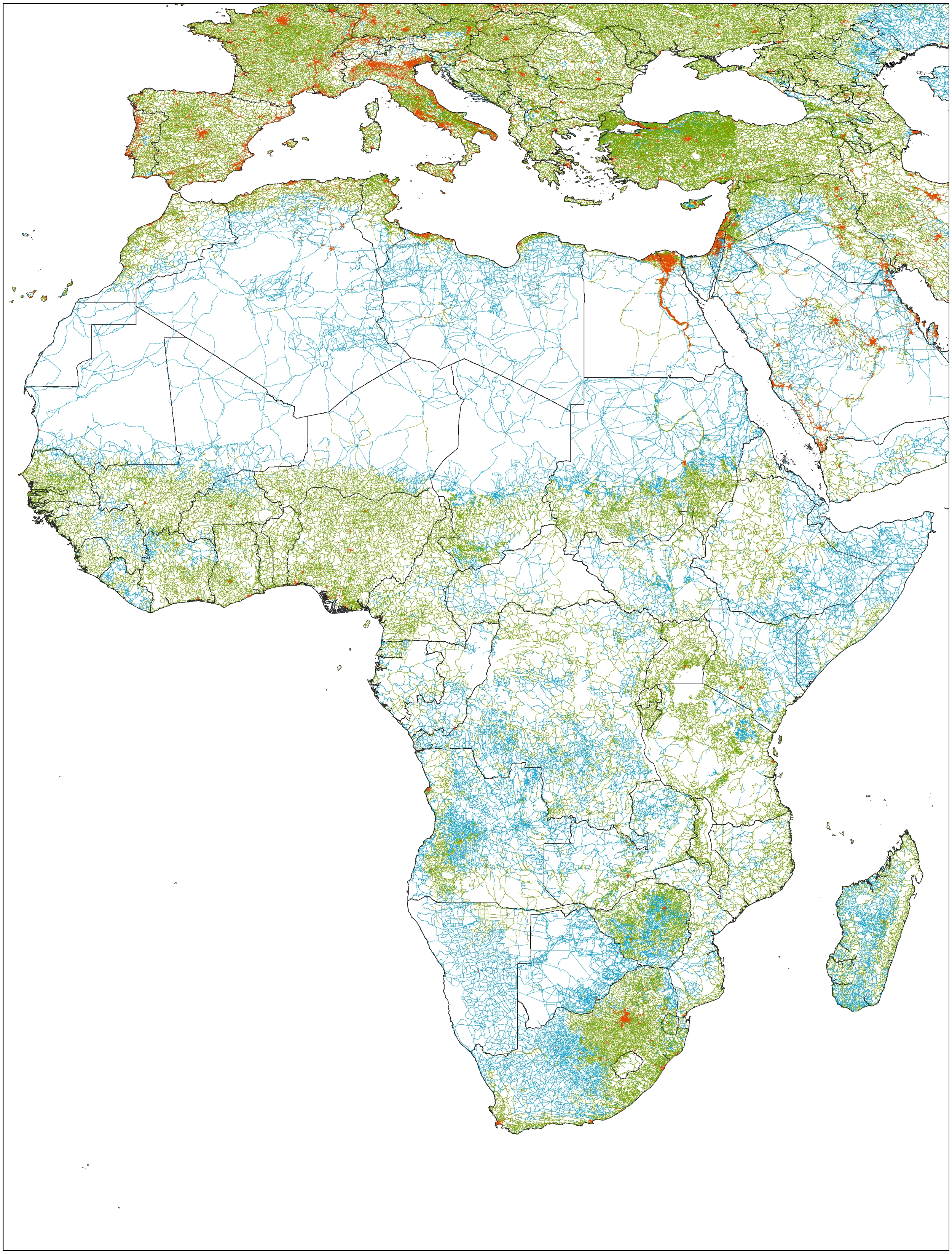

FIG. 9. GRN in Africa. Note the low density of roads compared to Europe.

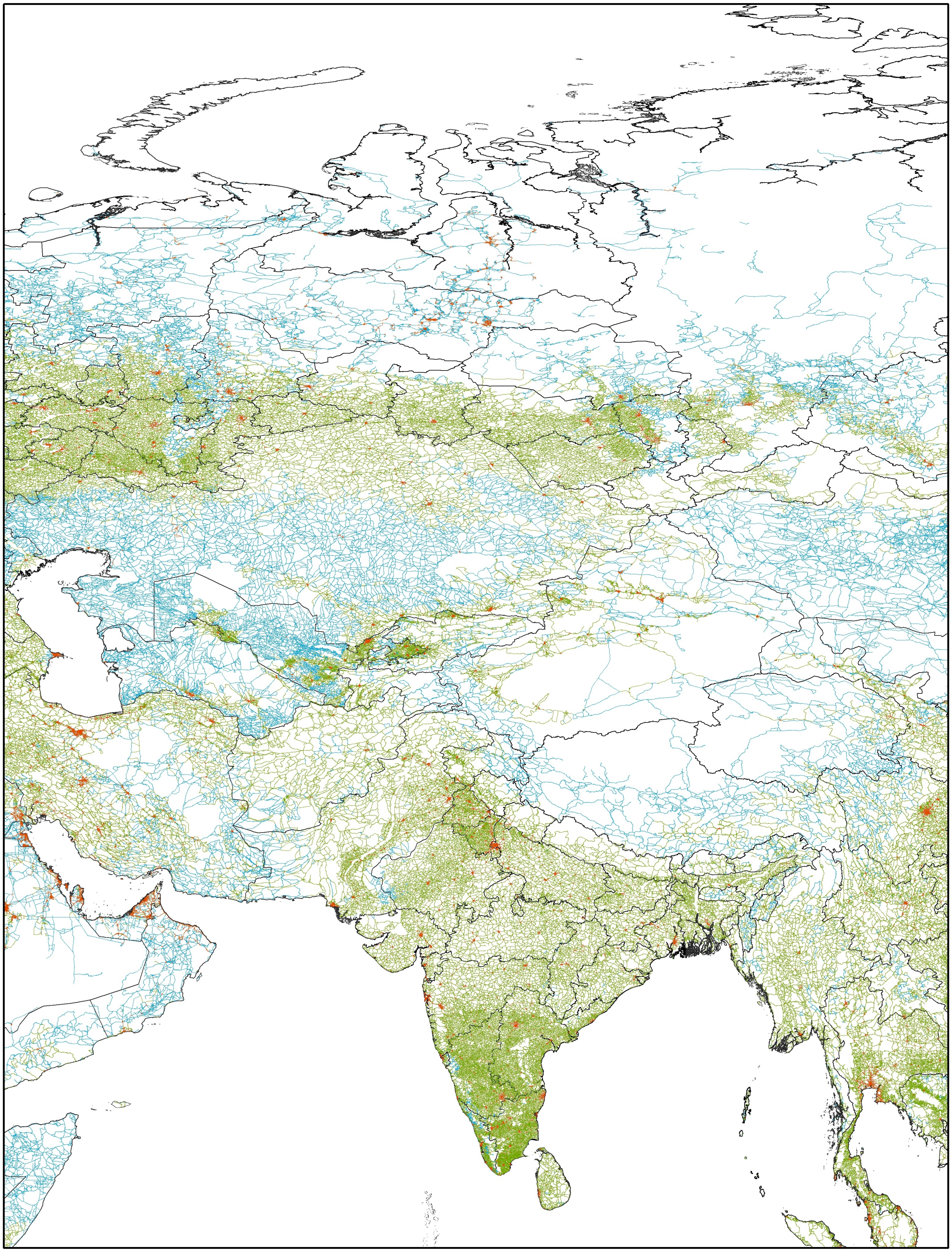

FIG. 10. Central Asia

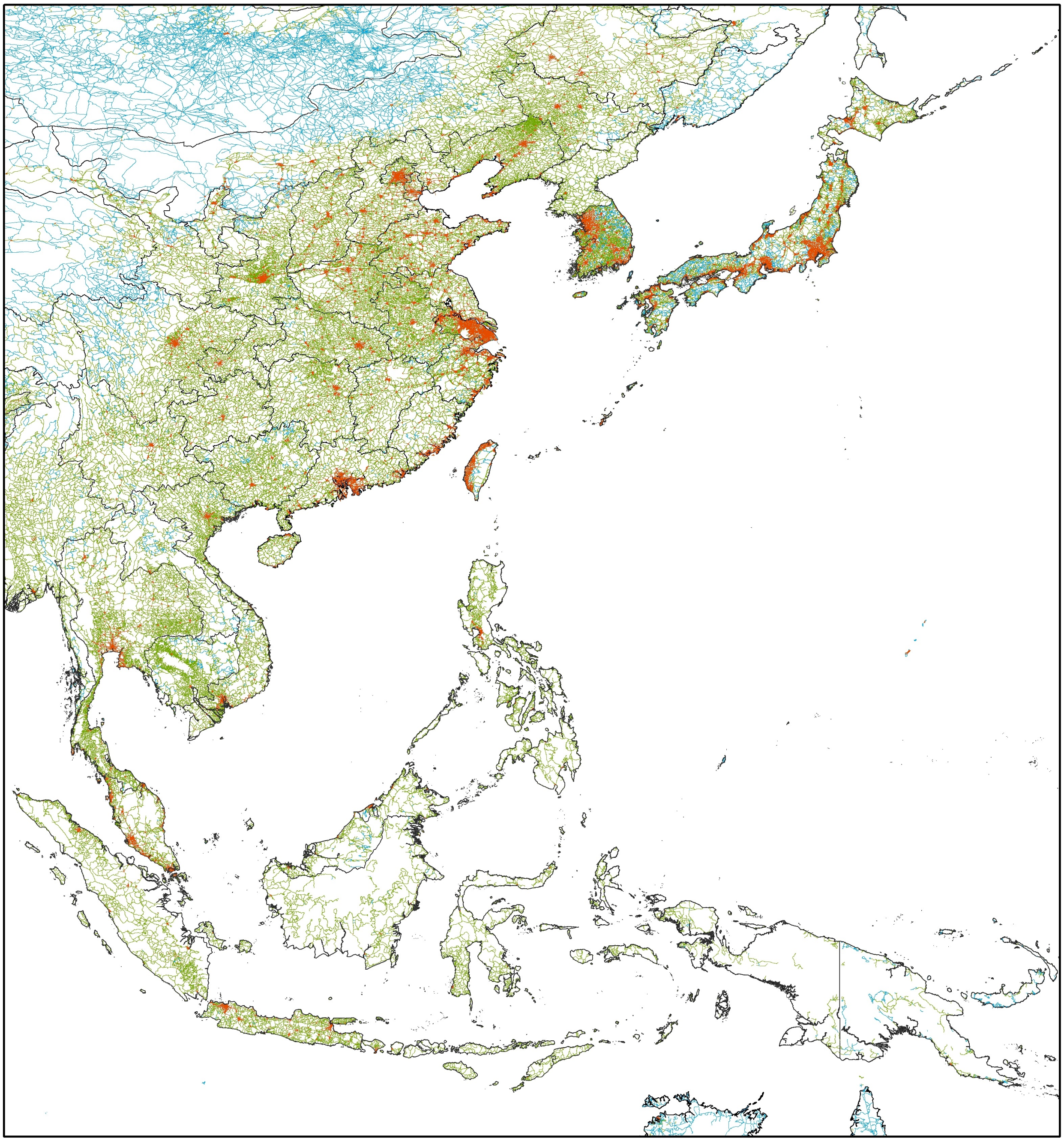

FIG. 11. East Asia

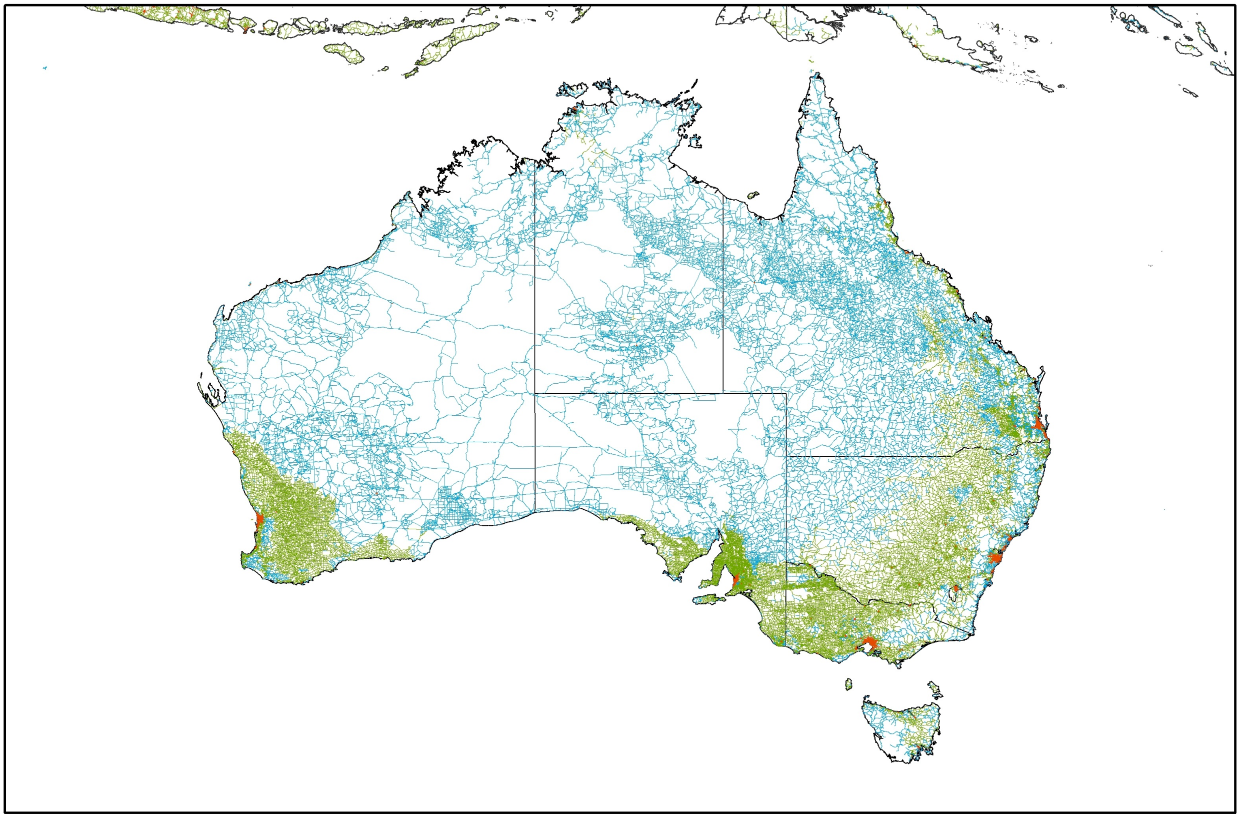

FIG. 12. Australia

REFERENCES

[1] C. D. Elvidge, P. Cinzano, D. Pettit, J. Arvesen, P. Sutton, C. Small, R. Nemani, T. Longcore, C. Rich, J. Safran, *et al.*, Int. J. Remote Sens. **28**, 2645 (2007).

[2] Q. Zhang and K. C. Seto, Remote Sens. Environ. **115**, 2320 (2011).

[3] G. F. Jenks, International yearbook of cartography **7**, 186 (1967).

[4] S. Fritz, L. See, I. McCallum, L. You, A. Bun, E. Moltchanova, M. Duerauer, F. Albrecht, C. Schill, C. Perger, *et al.*, Glob. Change Biol. **21**, 1980 (2015).
